# Common data elements for criminal legal system involvement for people who use drugs

**DOI:** 10.1186/s12954-025-01362-1

**Published:** 2025-12-11

**Authors:** Bradley Ray, Mia Christopher, Lissette M. Saavedra, Jessica Cance, David Seal, Rachel Gicquelais, Caitlin Conway, Dora Illei, Leo Beletsky

**Affiliations:** 1 RTI International, Research Triangle Park, NC USA; 2 Tulane University, New Orleans, LA USA; 3 University of Wisconsin-Madison, Madison, WI 53705 USA; 4 Northeastern University, Boston, USA

**Keywords:** Common data elements, Survey measures, Delphi, Persons who use drugs, Criminal-legal systems

## Abstract

The United States’ legal responses to substance use and addiction have led to frequent interactions between people who use drugs (PWUD) and criminal-legal systems. Interactions with policing, courts, detention facilities, and community supervision can shape health risk and disrupt access to services. Behavioral health and harm reduction organizations also frequently experience law enforcement interference on the program level. To better understand this structural determinant of PWUD health, we developed survey measures that assess PWUD’s contact with criminal-legal systems across 10 harm reduction studies. Using input from experts and leveraging the National Institutes of Health Research on Interventions for Stability & Engagement network, the survey captures a broad range of legal interactions. The resulting measures enhance comparability across studies, informing policies that address the complex relationship between criminal-legal involvement and health outcomes for PWUD, particularly in overdose prevention efforts.

## Introduction

In the United States (US) there are a multitude of reasons why people who use drugs (PWUD) may be frequently involved in criminal-legal systems, that mostly stem from the criminalization of drug use and possession [1–3]. At federal, state, and local jurisdictions law enforcement officers from a variety of agencies receive training and resources on how to detect, arrest, and incarcerate people who use specific substances [4]. However, involvement in US criminal-legal systems extends beyond these initial encounters into a spectrum of interactions with courts, carceral facilities, and community monitoring systems. Moreover, the practices aimed at detecting drug use once individuals are under supervision can result in repeat interactions and negative legal outcomes. All of these interactions can affect social determinants of health for PWUD by limiting access to employment, housing, healthcare and other social services [5–8].

North America remains in the midst of an overdose epidemic. Despite some recent declines [9], there have been over one million accidental drug overdose deaths in the past two decades [10–12]. Harm reduction strategies for drug use have emerged as a potential means for preventing overdose and other drug-related harms; these strategies focus on meeting PWUD where they are at to reduce harms [13]. For example, for more than 30 years syringe services programs (a harm reduction strategy for PWUD) have been a critical part of the United States public health strategy with a strong evidence-base for reducing HIV and bloodborne infection transmission through distributing new syringes and promoting safer injection [14–17]. However, harm reduction programs like these intersect with criminal-legal systems as they are providing services directly to PWUD [18–20]; program participants in one setting, and persons potentially violating the law in another.

Criminal-legal systems refer to the network of laws, institutions, and practices—police, courts, jails, prisons, and community supervision (probation and parole)—that define, enforce, adjudicate, and punish alleged crimes. While there is evidence that interactions with these systems shape health risks for PWUD, less is known about interactions with specific intercepts such as policing, jail, and community supervision. Prior research in this space has assessed experience with one of these criminal-legal systems intercepts or combined them to determine any contact with systems [21–26]. As research continues to examine efficacy of harm reduction strategies for PWUD, it is important to consistently measure interactions with criminal-legal systems among this population. With common data elements (CDE) both researchers and harm reductions practitioners can compare criminal-legal systems involvement among programs. In this study we describe the process of developing individual-level criminal-legal system (CLS) survey measures that could be administered to PWUD across multiple study settings. We describe the Delphi process and proposed use of survey measures among researchers and practitioners working with PWUD.

## Methods

The sparse evidence base on upstream contact with law enforcement suggests that participants receiving harm reduction services face numerous access barriers [20]. It also suggests that uneven distribution of such contact may impact some people more than others. [27] Conceptualizing contact with criminal-legal systems as a structural determinant of health is a novel field of inquiry [24]. Prior research on criminal-legal systems involvement among participants receiving harm reduction services is limited and are often provided anonymously [28] and has relied on self-report measures [20]. Prior studies have focused on interactions with police, not the eventual carceral outcome of that interaction, nor about surveillance by corrections agencies like probation (community supervision instead of further jail/prison) or parole (supervised release after prison). Thus, assessing criminal-legal systems involvement requires multiple items to capture various intercepts to determine the depth of involvement in these systems. For example, someone recently stopped by the police has a much different CLS interaction than someone who has been incarcerated and is now being monitored by probation or parole. Additionally, there are challenges to consider when measuring criminal-legal systems involvement, particularly around variation in criminal codes and definitions across jurisdictions, and concerns about self-reporting and the stigma that comes with systems involvement and community supervision. Therefore, we sought to develop items that could capture this nuance but without getting into jurisdiction specificity regarding criminal codes. For example, given variability across states we avoided items that ask about the type or charge level (e.g., felony or misdemeanor). Similarly, asking people about their police interactions regarding syringes can have drastically different outcomes depending on the state drug paraphernalia laws (e.g., in some states possession of a syringe is a felony and in others it is legal).

To develop CLS measures for PWUD we leveraged the National Institutes of Health (NIH) Helping End Addiction Long-Term® (HEAL) Initiative’s Research on Interventions for Stability & Engagement (RISE). This national network consists of the first 10 projects funded by the NIH to study and improve the effectiveness, implementation, and impact of existing and new harm reduction strategies [29, 30]. Since the development process was borne out these broader efforts to reduce overdose deaths [31], the CLS measures also ask about experiences with first responders at nonfatal overdose events. Finally, we sought to develop a brief number of items that could be integrated into studies that might be collecting data from PWUD, and especially as a part of overdose prevention efforts, or used by harm reduction practitioners. Therefore, to facilitate brevity the decision was made to collapse incarceration settings (jail and prison) and community supervision (probation and parole) despite these being distinct CLS with different implications for individuals.

### Process for developing CLS common data elements

RISE researchers employed an iterative, consensus-driven process to develop a set of common CLS measures, with careful attention to the diversity across the studies within the network. Study populations varied across geographic location, type of participant contact, population characteristics, harm reduction interventions being implemented, and the criminal legal setting and involvement [31]. Given the diversity of studies, it was critical that the CDE were appropriate for each study’s context and population. To inform the process, we sought expertise from the fields of criminal legal, law, harm reduction, public health, and PWUD. These experts ensured that the data elements represented a range of potential interactions that PWUD may have with criminal-legal systems, including those that occur during nonfatal overdose events involving law enforcement.

To reach consensus on the CDE, the RISE employed a Delphi method, a methodological approach that involves iteratives rounds that leverages subject matter expertise, discussion, and rounds of voting [23, 32]. This collaborative approach facilitated network-wide decision-making to identify data elements that were most relevant, sensitive, and useful for PWUD. The Delphi method includes generating of a list of data elements, forming a panel of experts, conducting multiple rounds of voting on data elements, and analyzing and disseminating the voting results until consensus is reached. To start this process, the RISE identified priority measure constructs – including CLS measures – at a network-wide, in-person RISE meeting. Following this, research projects submitted their draft survey instruments to the Coordination Center (RTI International) for review for measures of interest and cross-study alignment.

RISE common data element development was conducted through seven iterative rounds, each following a standardized process. Each iteration involved four steps: (1) candidate measures were collated and distributed for review by a Delphi panel composed of Principal Investigator (PI), study team members, and affiliated community representatives from each research project, (2) sites then evaluated the measures and provided structured feedback, (3) this feedback, both quantitative (voting and rating on a scale) and qualitative (open-text responses), was compiled and analyzed to inform measure refinement, and (4) a revised set of CDEs was subsequently retribution in the next iteration. This process was repeated seven times until consensus was reached across all research projects. Each RISE research project was allotted one vote, typically cast by the study PI; however, when the PI was unavailable, another research team member was designated to vote, ensuring representation from all projects during each meeting.

## Results

### Expert-led prioritization and refinement of CLS constructs

Ensuring that measures were appropriate for each research study and its specific population was critical, particularly in the context of criminal legal system involvement for PWUD. For instance, because drug paraphernalia laws differ significantly by state, network-level items needed to be worded to ensure applicability across jurisdictions. In some cases, measures required modification or were designated as optional to protect participants given the local legal context and the point along the Sequential Intercept Model at which the study team engaged with participants [33]. Additionally, certain were excluded when they were not relevant or appropriate for specific study populations.

To guide the development of CLS CDEs, each research project engaged community experts and people who use drugs through study-specific Community Advisory Boards. Between iterations by the Data Harmonization Workgroup, research teams facilitated community review of proposed measures to ensure appropriate language, relevance, safety, accuracy, and utility. Feedback from community advisors was integrated into each round of revisions to ensure the measures accurately reflected the lived experience of criminal legal system involvement among people who use drugs. A subgroup of the RISE Data Harmonization Workgroup also convened experts with CLS expertise. This subgroup played a central role in identifying and refining key constructs for inclusion in the network-wide CDES. They initially identified three primary domains that the network should prioritize for involvement of PWUD with the criminal-legal system: (1) law enforcement contact (e.g., stops, searches, arrests), (2) incarceration experiences (jail, prison, detention), and (3) post-release supervision (e.g., probation, parole). While post-release and incarceration experiences were considered relatively well covered in existing instruments, the subgroup noted the need for further development of items related law enforcement contact, especially in the context of PWUD. There was an emphasis on capturing a broad range of interactions with criminal-legal systems—not just arrests—to accurately reflect participant experiences. The subgroup also prioritized inclusion of measures related to the involvement of child protective services and other child welfare systems. Members highlighted the need to document how decisions around child custody and family separate are influenced by participants’ interactions with the criminal legal system, including stigmatization of harm reduction services like syringe exchange program participants.

### Collaborative sourcing and iterative consensus on core CLS measure

***s***Recognizing the complex and sensitive nature of these topics, the subgroup made a concerted effort to engage members of the broader Data Harmonization Workgroup and RISE study teams with expertise in the criminal-legal systems. The group began by identifying priority constructs for network-wide measurement. They then compiled example measures from existing studies—including the Justice Community Opioid Innovation Network, the Rural Opioid Initiative, and the Seek, Test, Treat, and Retain initiative. Once all relevant measures were collected, the subgroup reconvened to review and prioritize items. Selected measures were then presented to the Data Harmonization Workgroup for consideration and incorporated into a subsequent iteration for structured rating, voting, and qualitative feedback.

Through deliberation with the CLS experts, approximately 70 candidate CLS measures were initially presented to the Data Harmonization Workgroup. Over seven iterative rounds, the workgroup refined and narrowed the list, identifying eight CLS measures to capture criminal legal involvement among people who use drugs across the RISE (Table 1). Four core measures—assessing self-reported experiences of police contact, arrests, jail and prison stays, and probation and parole status within the past 30 days—were adopted by all research projects to enable cross-study analysis. The remaining four measures, which included items on police encounters following an overdose and perceived likelihood of arrest en route for harm reduction services, or for paraphernalia possession, were recommended by not required due to difference in study populations and contexts. The selected measures align with recommendations from the CLS subgroup and are relevant to understanding the legal system experience of people who use drugs across the network.

**Table 1:** Common data elements for criminal-legal system involvement for people who use drugs

Variable	Measure	Response options
Police contact	In the past 30 days, how many times were you stopped by the police (even if it didn’t lead to arrest or further legal consequences)?	a. _______ (# of times) b. Don’t know c. Prefer not to answer
Arrest	In the past 30 days, how many times have you been arrested?	
Jail/Prison	In the past 30 days, how many nights have you been held overnight in jail or prison?	
Probation/Parole	At any time in the past 30 days have you been on probation or parole?	
Police encounter after overdose (self)	Have any of these things ever happened to you as a result of an overdose?	
	a. Had a warrant run	a. Yes b. No c. Don’t know d. Prefer not to answer
	b. Got a ticket or citation	
	c. Got arrested	
	d. Experienced problems with Child Protective Services (CPS)	
	e. Experienced problems with you housing or landlord (like got evicted)	
Police encounter after overdose (acquaintance)	Have any of these things ever happened to someone you know as a result of an overdose?	
	a. Had a warrant run	a. Yes b. No c. Don’t know d. Prefer not to answer
	b. Got a ticket or citation	
	c. Got arrested	
	d. Experienced problems with Child Protective Services (CPS)	
	e. Experienced problems with you housing or landlord (like got evicted)	
Arrest likelihood (harm reduction services)	How likely do you think it is that you could get arrested en route to or from harm reduction services?	a. Very unlikely b. Somewhat unlikely c. Somewhat likely d. Very likely e. Don’t know d. Prefer not to answer
Arrest likelihood (paraphernalia)	How likely do you think it is that you could get arrested for paraphernalia possession if you are stopped by the police when you are carrying new, unused syringes?	

## Discussion

The development of CDEs for CLS involvement is a crucial step in enhancing the rigor of research on harm reduction and other strategies for PWUD [34]. By standardizing CLS measures across studies, researchers can improve comparability, allowing for more robust meta-analyses and longitudinal assessments. Standardized data can also strengthen replicability, ensuring that findings are consistent across diverse geographic and policy contexts. Moreover, data-driven advocacy efforts require reliable and comparable metrics; by implementing CLS CDEs, harm reduction advocates and policymakers can use empirical evidence to push for policy reforms that minimize the negative health and social consequences of criminal-legal involvement for PWUD.

To fully realize the potential of CLS CDEs, future research should focus on expanding their adoption, including additional research networks. This includes collaborations with public health agencies, criminal-legal systems researchers, and social service organizations that work directly with PWUD. Harmonized CLS measures developed with input from people who use drugs and CLS research experts can improve visibility into systemic harms caused by CLS involvement, support design of more responsive programs and policies, and ensure the public health response is grounded in the lived experiences of those most impacted. Additionally, integrating CLS CDEs with health and social service datasets will provide a more comprehensive understanding of how criminal-legal involvement interacts with healthcare access, housing stability, employment, and other social determinants of health. This cross-sectoral approach can illuminate systemic barriers and inform evidence-based interventions. Moreover, encouraging use of the RISE CLS measures will enhance the ability to generate actionable insights that inform public health interventions and policy changes.

There are several limitations and lessons learned from the development of these CLS measures. These items have not yet been validated, which might limit uptake. There may be differences in CLS structures across jurisdictions that result in variations in participant comprehension of terminology and recall issue around retrospective CLS experiences. In terms of lessons learned, we found it important to have transparency in background and expertise as part of the consensus-building process. We also found that it was important to allow flexibility for site-specific adaptations. For example, some studies might consistently be asking about the prior 3 months, rather than 30 days, requiring changes to the CLS measures. Finally, we heard from our expert panelists that there is going to be need for updates to the CDE as policies around drug enforcement evolve. Despite these limitations, the CLS measures developed for the RISE provide an incredible starting point for further research. This should include rigorously validating the CLS CDEs through multi-site studies that include cognitive interviewing with PWUD, test–retest reliability, and measurement invariance analyses across jurisdictions, languages, and key subgroups. Convergent, criterion, and predictive validity can be evaluated by linking self-report to criminal records where feasible and by testing associations with downstream outcomes and policy shifts (e.g., paraphernalia or possession reforms). Implementation studies can assess mode and recall-period effects (self-admin vs. interviewer; 30- vs. 90-day), refine wording/scoring, and confirm that shortened forms maintain accuracy for routine use by harm reduction programs.

## Conclusion

The integration of CLS CDE into harm reduction research is a critical step toward improving data quality, study comparability, and policy advocacy. By adopting expert-driven, standardized approaches, researchers can build more cohesive evidence on the impact of CLS involvement on PWUD, ultimately strengthening interventions. Through data-driven insights and collaborative efforts, standardized CLS data can play a pivotal role in improving the lives of PWUD and advancing strategies that reduce overdose and other drug-related harms.

## Data Availability

Not applicable.
